# Transcatheter aortic valve replacement over age 90: Risks vs benefits

**DOI:** 10.1002/clc.23310

**Published:** 2019-12-16

**Authors:** Christos Galatas, Jonathan Afilalo

**Affiliations:** ^1^ Jewish General Hospital McGill University Montreal Quebec Canada

**Keywords:** valvular < surgery/adult, valvular heart disease, aging and the cardiovascular system

## Abstract

As the population ages, clinicians will encounter a growing number of nonagenarians suffering from severe aortic stenosis who may be candidates for transcatheter aortic valve replacement (TAVR). By virtue of a healthy survivor effect or a referral bias, these patients may paradoxically have greater resilience and fewer comorbidities than their octogenarian counterparts. They tend to, on average, tolerate the TAVR procedure quite well with low in‐hospital and 1‐year mortality rates of 5.5% and 23%, respectively. Appropriate patient selection should consider individualized estimates of procedural risk, potential for functional recovery and for improved quantity and quality of life. Frailty is much more revealing than chronological age, and it can be measured by brief tools such as the Essential Frailty Toolset. Ultimately, the process of shared decision‐making is paramount to ensure that the course of action is patient‐centered and balances the procedure's expected risks and benefits with the nonagenarian's preferences and values.

## 1 INTRODUCTION

Calcific aortic stenosis (AS) is the most common form of acquired valvular heart disease in older adults.^1^ The pathophysiology of calcific AS is closely connected to the aging process; with inflammaging, calcification, and repetitive mechanical stress being among the driving mechanisms.^2^ An estimated 17% of nonagenarians will develop at least mild AS over the course of their lifetime.^3^ By 2050, the number of nonagenarians is expected to quadruple to >8 million in the United States,^4^ and given that there is no proven therapy for the prevention of AS, the number of “oldest old” patients suffering from this disease is expected to mirror the demographic population trends and rise exponentially. When AS becomes severe it is often associated with debilitating symptoms, reduced functional capacity, hospitalizations, and heart failure eventually leading to death. Prognosis is poor in the absence of aortic valve replacement, and historically nonagenarians were excluded from this surgery due to the higher procedural risks and lower perceived benefits.

Transcatheter aortic valve replacement (TAVR) has emerged as a therapeutic option for severe symptomatic AS in patients at high surgical risk^5^, ^6^ as well as intermediate and low surgical risk.^7^, ^8^, ^9^ Nonagenarians were not excluded from this procedure, to the contrary, initial TAVR trials targeted patients that were deemed too old or frail to undergo surgery. Early experiences indicated that these patients could undergo the minimally invasive TAVR procedure with acceptable risk. Since that time, the number of TAVRs performed in nonagenarians has progressively increased, accounting for one out of seven TAVR procedures in the United States between 2011 and 2014.^10^ As nonagenarians are increasingly referred for consideration of TAVR, clinicians are tasked to identify the “good 90‐year‐old” who will likely tolerate the procedure and derive meaningful benefits—a forecast that is imperfect but informed by objectifiable features. The goal of this article is to review the literature on TAVR in nonagenarians and to provide guidance for individualized patient‐centered decision making.

## 2 THE NONAGENARIAN PHENOTYPE

According to actuarial life tables in the United States, the average life expectancy at birth is 76.0 years for men and 80.1 years for women.^11^ Having surpassed this life expectancy by more than a decade, nonagenarians have effectively overcome competing risks and declared themselves to be more resilient to stressors encountered during the course of their lives. Given this self‐selection, the average remaining life expectancy at age 90 is not trivial; calculated to be 4.1 years for men and 4.9 years for women. Otherwise said, the annual probability of death at age 90 is only 16% for men and 13% for women, respectively.

One might assume that nonagenarians would have higher rates of comorbidities as compared to their relatively younger counterparts. However, a “healthy survivor” effect has been observed whereby nonagenarians—by virtue of their achieved survival—have paradoxically lower rates of comorbidities. Still, most nonagenarians with severe AS will likely have at least one significant comorbidity. In a Spanish study of nonagenarians with severe AS managed both with TAVR and conservatively, the most common comorbidity was chronic kidney disease, which was present in 70% of the cohort.^12^ Other comorbidities present in at least 10% of the cohort were diabetes mellitus (32%), myocardial infarction (16%), dementia (13%), and previous stroke (11%). The mean Charlson Comorbidity Index score was 3.2 and only 32% of the cohort had a low comorbidity burden; a high comorbidity burden was associated with increased 1‐year mortality. In particular, end‐stage renal disease and oxygen‐dependent lung disease have been associated with markedly increased risks of mortality and major morbidity.^13^

Frailty, defined as a diminished capability to recover from pathological or iatrogenic stressors due to cumulative age‐related impairments, is a key consideration in nonagenarians. Impairments may be broadly categorized as physical (loss of muscle mass and strength, ie, sarcopenia), cognitive, and psychosocial. Numerous scales have been developed to operationalize the assessment of these impairments, with the prevalence and prognostic impact of frailty varying non‐negligibly depending on the scale used. The FRAILTY‐AVR study was conducted to compare the value of seven different frailty scales in >1000 patients 70 to 99 years of age undergoing transcatheter or surgical aortic valve replacement. In this study, the Essential Frailty Toolset (EFT) was found to be most predictive of 1‐year mortality (OR 3.72, 95% CI 2.54‐5.45) and worsening disability (OR 3.29, 95% CI 1.73‐6.26).^14^ The EFT is scored 0 to 5 based on lower extremity strength, cognitive function, hemoglobin, and serum albumin. Frailty has also been shown to be predictive of short‐term mortality, major bleeding, discharge to skilled‐nursing facilities, and worsening quality of life following TAVR.^15^

Several age‐related cardiac impairments have been described in nonagenarians.^16^ Even in the absence of overt cardiovascular disease, nonagenarians have smaller LV cavities, more concentric left ventricular remodeling and hypertrophy, diastolic dysfunction, and mitral annular calcification. These structural changes become more severe in response to chronic pressure overload from AS, and they may introduce technical complexity when deploying a transcatheter valve. Nonagenarians undergoing TAVR are also more likely to present with multi‐valve disease^3^; with 15%‐64% exhibiting concomitant moderate or severe mitral regurgitation^17^, ^18^, ^19^, ^20^, ^21^, ^22^, ^23^, ^24^, ^25^, ^26^, ^27^ —a finding associated with a 2‐fold increase in mortality following TAVR.^12^ Another common yet concerning finding in nonagenarians is pulmonary hypertension; with 21%‐44% exhibiting systolic pulmonary arterial pressures >60 mmHg.^19^, ^20^, ^21^, ^22^, ^24^, ^26^, ^27^

## 3 TRANSCATHETER AORTIC VALVE REPLACEMENT IN NONAGENARIANS

The evidence for TAVR in nonagenarians stems primarily from cohort studies and case series (with >30 such studies published since 2012; Table 1) and also from subgroup analyses of randomized clinical trials. From this body of evidence, the patient characteristics and relative risks and benefits of TAVR have been compared across age groups. Nonagenarians undergoing TAVR are more likely to be women, which is not surprising considering that nonagenarian women outnumber nonagenarian men by 3‐to‐1 in the general population. Nonagenarians undergoing TAVR are less likely to present with comorbid coronary artery disease, peripheral arterial disease, diabetes mellitus, and multiple chronic conditions.^10^, ^21^, ^24^, ^27^, ^28^, ^29^, ^30^, ^31^, ^32^, ^33^ These differences can be explained by the healthy survivor effect, the clinician's cognitive bias to be conservative in nonagenarians with significant comorbidities, or a combination of the two.

**Table 1:** Reviewed studies

Study	Design	N (%) 90+	STS‐PROM	Procedural success	Major vascular	Major bleed	Stroke	30‐d mortality	1‐y mortality
Barth 2019	MC, P	68 (7)	NA	84%	17.6%^a^	11.8%	NA	10.3%^a^	NA
Stehli 2019	MC, P	71 (12)	5.7%	96%	7.0%	0.0%	1.4%	0.0%	11.4%
Vlastra 2019	MC, P	882 (7)	9.9%	NA	NA	8.1%^a^	3.0%^a^	9.9%^a^	NA
Yokoyama 2019	MC, P	94 (12)	8.3%	NA	19.1%^a^	4.0%	6.4%	2.1%	NA
Stamou 2019	SC, R	148 (100)	NA	NA	8.8%	37.2% (T)	2.7%	6.8% (H)	19.0%
Scholtz 2018	SC, P	82 (8)	8.5%	98%	4.9%	NA	3.6%	9.8%^a^	30.9%
Vendrik 2018	SC, P	47 (8)	8.0%	79%	10.6%^a^	6.4%	6.4%	2.1% (H)	NA
Ichimoto 2018	SC, R	17 (20)	12.3%	NA	11.8%^a^	5.9%	0.0%	0.0%	NA
Doshi 2018	MC, R	1163 (33)	NA	NA	4.5%	35.0% (T)	3.4%	6.0% (H)^a^	NA
Elgendy 2018	MC, R	5840 (100)	NA	NA	3.3%	28.3% (T)	3.3%	6.6% (H)	NA
Miura 2017	SC, P	25 (22)	10.0%	96%	8.0%	4.0%	4.0%	0.0%	8.4%
Okoh 2017	SC, R	75 (100)	9.6%	NA	NA	NA	0.0%	6.7%	NA
McNeely 2017	MC, R	3531(19)	NA	NA	NA	34.2%^a^	1.8%	8.4%^a^	25.4%^a^
Mendiz 2017	MC, R	33 (100)	11.1%	97%	9.1%	18.2%	0.0%	9.1%	NA
De Biasi 2017	SC, P	25 (100)	10.2%	NA	0.0%	36.0%	0.0%	0.0%	17.0%
Biancari 2017	MC, P	80 (100)	NA	NA	3.8%	27.5% (T)	0.0%	6.3%	NA
Zack 2017	MC, R	695 (100)	NA	NA	11.9%	33.7% (T)	3.6%	6.5% (H)	NA
Penkalla 2016	SC, R	40 (100)	24.2%	NA	5.0%	10.0%	7.5%	10.0%	41.4%
Escarcega 2016	SC, R	107 (16)	12.1%	NA	13.1%	13.1%	1.9%	5.7% (H)	25.0%
Arsalan 2016	MC, P	3773 (16)	9.2%	NA	1.0%^a^	8.1%^a^	2.7%^a^	8.8%^a^	24.8%^a^
Greason 2015	SC, R	46 (100)	NA	NA	21.7%	NA	2.2%	4.7% (H)	15.4%
Kayatta 2015	SC, R	95 (100)	14.5%	NA	NA	2.1%	2.0%	3.2%	24.5%
Thourani 2015	MC, P	531 (100)	NA	75%	6.2%	NA	2.1%	7.2%	NA
Abramowitz 2015	SC, R	136 (19)	11.0%	93%	4.4%	5.9%	2.9%	2.9%	NA
Mack 2015	MC, R	90 (100)	11.6%	NA	NA	NA	2.2%	11.1%	30.0%
Murashita 2014	SC, R	26 (100)	10.3%	NA	23.1%	NA	3.9%	3.9%	NA
Pascual 2014	MC, R	19 (100)	NA	NA	10.5%	NA	NA	5.3% (H)	NA
Yamamoto 2014	MC, P	346 (15)	NA	97%	5.8%	5.5%	4.0%	11.3%	NA
Noble 2014	SC, P	23 (100)	8.7%	74%	0.0%	13.0%	4.3%	8.7%	NA
Verouhis 2014	SC, R	29 (100)	6.2%	100%	0.0%	0.0%	3.4%	0.0%	10.7%
Akin 2012	SC, R	11 (100)	25.3%	100%	9.1%	18.2%	18.2%	27.3%	NA
Yamamoto 2012	SC, P	26 (19)	13.4%	100%	19.2%	34.6%	3.8%	15.4%	NA

a Denotes studies that reported an increased risk in patients ≥90 years of age compared to <90 years of age (not tested in all studies).

Abbreviations: H, in‐hospital; MC, multicenter; NA, not available; P, prospective; R, retrospective; STS‐PROM, Society of Thoracic Surgeons Predicted Risk of Mortality; SC, single center; T, transfusion of packed red blood cells.

TAVR can be performed in nonagenarians with high procedural success rates and acceptable in‐hospital, 30‐day, and 1‐year mortality rates. In most studies, the procedural success rate was >95% and the absolute stroke rate was ≤4% above age 90, with no consistent effect‐modification by age. In a meta‐analysis by Sun et al,^34^ the major bleeding rate was similar above and below age 90, with a relative risk of 1.17 (95% CI 1.04 to 1.32). Conversely, the vascular complication rate was mostly shown to be increased above age 90, especially when nonfemoral access techniques were used. In a sub‐study from the FRAILTY‐AVR study, nonfemoral access was associated with higher 30‐day mortality in frail patients (odds ratio 3.91, 95% CI 1.48 to 10.31) whereas this was not the case in robust patients (OR 1.29, 95% CI 0.34 to 4.94).^35^ While this comparison of femoral and nonfemoral access routes is clearly not randomized, most sources support the notion of higher risks in nonfemoral TAVR^13^, ^30^, ^35^, ^36^, ^37^ and favor femoral TAVR in older patients whenever possible.

**Figure 1:**
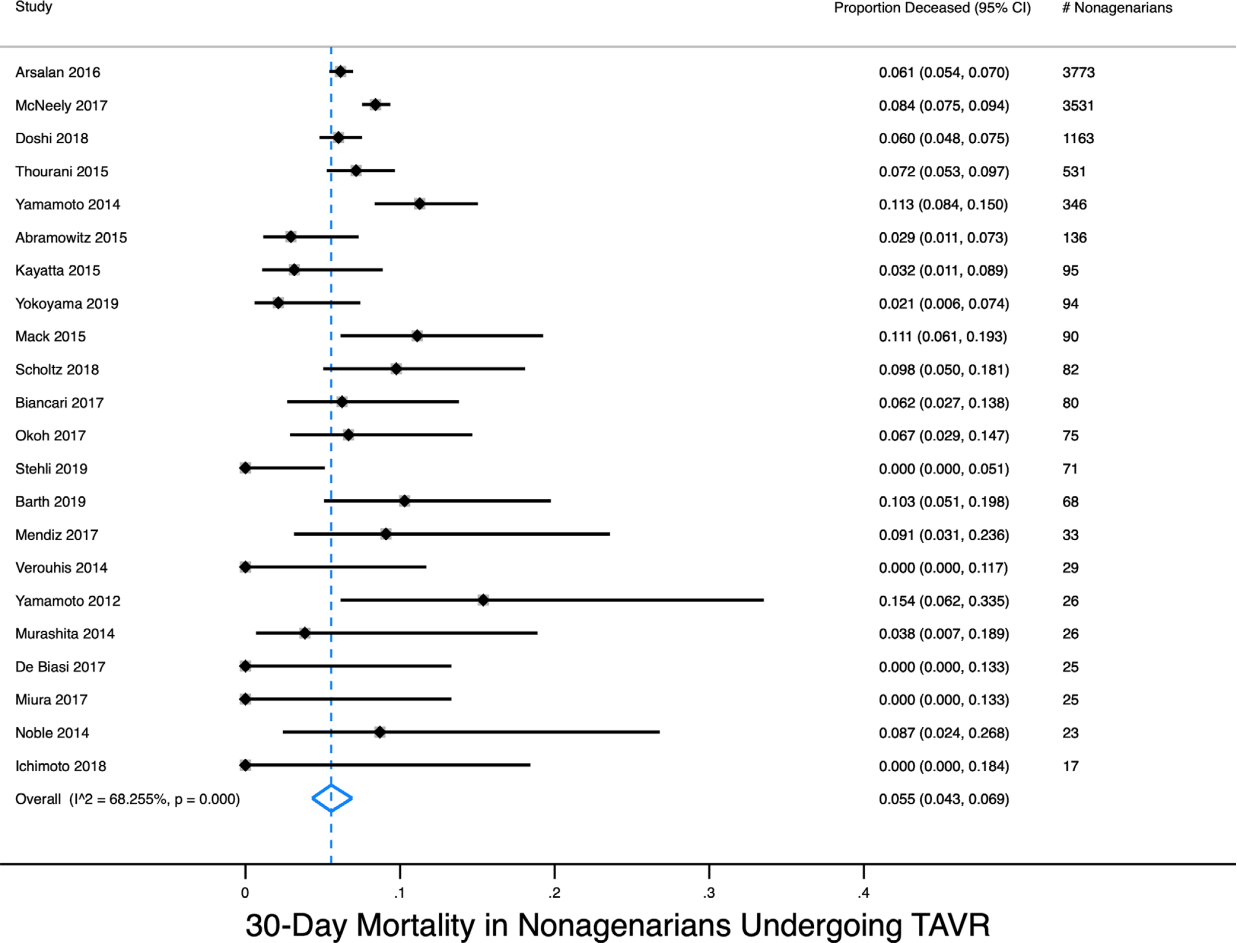
Meta‐analysis of 30‐d mortality in nonagenarians undergoing TAVR

**Figure 2:**
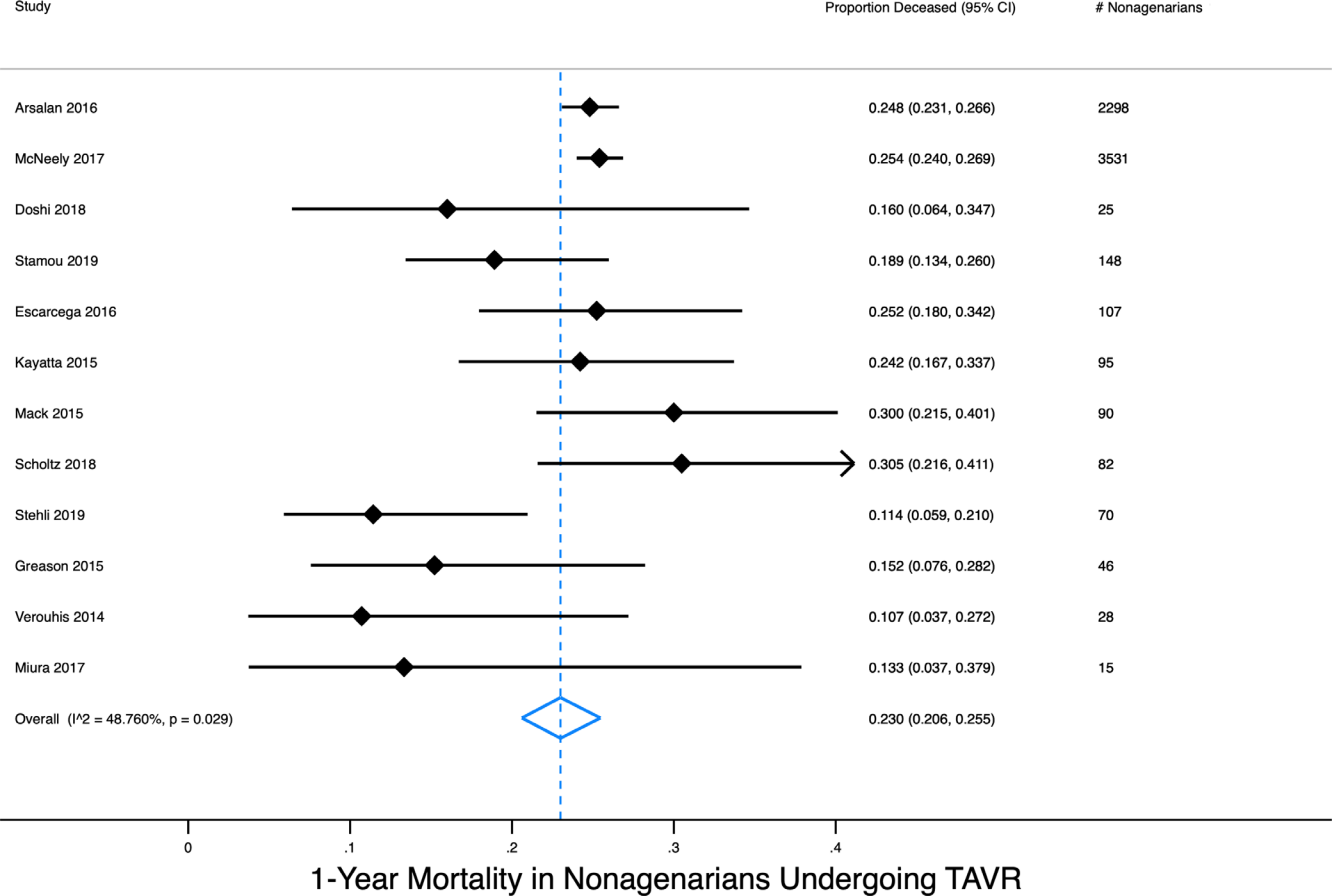
Meta‐analysis of 1‐y mortality in nonagenarians undergoing TAVR

Regarding survival, we performed a random‐effects meta‐analysis of observational studies that had reported deaths following TAVR in patients aged 90 years and above. In 22 studies encompassing 10 339 nonagenarians, the pooled 30‐day mortality rate was 5.5% (95% CI 4.3% to 6.9%) (Figure 1). In 12 studies encompassing 6535 nonagenarians, the pooled 1‐year mortality rate was 23.0% (95% CI 20.6% to 25.5%) (Figure 2). Interestingly, this 1‐year mortality is not drastically different than that of nonagenarians in the general population, which is approximately 15%. In studies comparing patients above and below age 90, the risk of mortality was slightly increased in some studies and no different in others.^10^, ^19^, ^21^, ^24^, ^27^, ^28^, ^29^, ^30^, ^32^, ^33^, ^38^, ^39^, ^40^ This inconsistency is at least partially attributable to variable adjustment for age‐related confounders.

Another reason for the inconsistent association between age and adverse outcomes is that chronological age is a flawed surrogate for biological aging and cumulative effects of clinical and subclinical impairments throughout the body. Frailty has been said to be a better indicator of biological aging and accumulated deficits. Nine studies measured frailty by at least one objective metric such as the Clinical Frailty Scale or the 5‐m gait speed test.^8^, ^10^, ^17^, ^28^, ^30^, ^33^, ^39^, ^41^, ^42^ Frailty was consistently found to be predictive of mortality and major morbidity after adjusting for comorbid conditions and cardiac status. Interestingly, the comparison of frailty metrics between nonagenarian and octogenarian patients was similar or only minimally increased in magnitude. In the FRAILTY‐AVR study, the mean EFT score was 1.8 ± 1.2 in septuagenarians, 2.0 ± 1.2 in octogenarians, and 2.3 ± 1.3 in nonagenarians (unpublished data). Disability for basic activities of daily living as measured by the Katz score was also found to be predictive of mortality and major morbidity.^8^

Regarding health‐related quality of life, two studies compared the effect of TAVR in patients above and below age 90.^10^, ^36^ In a multicenter prospective cohort study by Arsalan et al, 81% of nonagenarians had significant symptoms as evidenced by baseline NYHA class of III/IV with an average Kansas City Cardiomyopathy Questionnaire (KCCQ) score of 42. Post‐TAVR KCCQ scores improved to the same value of 75 in nonagenarians and non‐nonagenarians. Nonagenarians benefited from low heart failure readmission rates at 30 days and 1 year (5% and 15%) similar to their younger counterparts. In a study by Thourani et al, post‐TAVR KCCQ scores improved to the same value of 72 in nonagenarians and non‐nonagenarians, and other quality of life indices such as the Short Form‐12 and EuroQol‐5D improved comparably across age groups. Neither of these studies evaluated longitudinal changes in KCCQ scores among medically managed nonagenarians, although these would not be expected to improve given the evidence from previous studies and the progressive nature of severe AS.

Limitations of the data deserve consideration. Firstly, the nonagenarian patients in the reviewed studies may have been highly selected. This is suggested by their relatively lower burden of comorbidities, and by clinicians' inherent trepidation when it comes to performing invasive procedures above age 90. It is unclear how widely the evidence can be extrapolated to all nonagenarians with severe AS. Secondly, the experiences in these studies reflect ideal conditions of first procedures via mostly femoral access routes in higher‐volume centers. Outcomes associated with complex TAVR procedures (valve‐in‐valve, nonfemoral vascular access) or those in lower‐volume centers may be less encouraging. Thirdly, objective measures of frailty were not consistently reported or systematically used to guide patient selection. Frailty may be especially useful in this subgroup of patients given the expected dissimilarity in risks‐to‐benefits for frail 90‐year‐olds as compared to robust 90‐year‐olds. Notwithstanding these limitations, we can conclude that TAVR is a safe and effective therapeutic option for many nonagenarians—if carefully selected.

## 4 SELECTING THE RIGHT NONAGENARIAN PATIENT FOR TAVR

Selecting the appropriate nonagenarian for TAVR involves forecasting short‐term risks, mid‐term recovery, and long‐term benefits of the procedure. In the short‐term, is the patient at risk for a major procedural complication? The STS Risk calculator (http://riskcalc.sts.org/) was developed for SAVR but is still appropriately used as a benchmark to stratify candidates for TAVR.^38^ The ACC/STS TAVR In‐Hospital Mortality Risk Calculator was developed and calibrated for TAVR and accepts ages up to 100 (http://tools.acc.org/TAVRRisk/). Absolute complication rates remain low on average, and comorbidity‐complication dyads may be more telling to guide decisions, that is, chronic kidney disease and risk of acute kidney injury, protruding aortic atheroma and risk of stroke, bulky aortic valve calcification and risk of coronary occlusion, peripheral arterial disease and risk of vascular complications—particularly if femoral access is not feasible. Escalation of procedural complexity (through nonfemoral access or concomitant interventions) is not trivial in nonagenarians and can materially shift the delicate balance of risks.

In the mid‐term, is the patient likely to recover from the procedure and return home within a reasonable timeframe? Conversely, the less‐fortunate patient suffers a vicious cycle of ongoing deconditioning, prolonged hospitalization, and discharge to another healthcare facility. Attributes that may markedly impede or slow down the recovery process include: physical frailty and poor mobility, low social support, active depression. Physical frailty can be assessed with the chair rise and 5‐m gait speed tests, among others.^43^, ^44^

In the long‐term, is the patient likely to benefit from the implanted TAVR in terms of both quantity and quality of life? Irrespective of the technical success of the TAVR procedure, attributes that may preclude any improvement in functional capacity include: advanced dementia, bed‐ or wheelchair‐bound, cachexia or severe sarcopenia, and disability for all or most basic activities of daily living. Comorbidities that may preclude meaningful improvements in life expectancy include: end‐stage dialysis‐dependent renal disease, oxygen‐dependent lung disease, cirrhosis, and advanced cancer. The underlined A‐B‐C‐D‐E mnemonic represents the attributes and comorbidities that should raise serious concerns about the futility of TAVR. Lastly, older patients often present with fatigue or other nonspecific symptoms, and it is important to objectify the functional limitations attributable to AS (and thereby improvable by TAVR). Functional capacity can and should be objectively assessed with the 6‐minute walk test.

Once the information for forecasting risks and benefits has been acquired and analyzed, the clinician can better present the therapeutic options to the patient and assist them in reaching an informed decision that is in‐line with their preferences and values^45^ (Table 2). The options may include proceeding with TAVR, proceeding with balloon aortic valvuloplasty, continuing medical management of symptoms and ideally referring to a multi‐disciplinary heart failure clinic, or transitioning to a palliative care plan.^46^ Balloon aortic valvuloplasty may be chosen to observe the patient's potential for functional recovery and determine eligibility for TAVR in the future. A good TAVR candidate should exhibit meaningful improvements in physical frailty and functional capacity 3 to 6 months after valvuloplasty (ie, >1 point in the short physical performance battery and >50 m in the 6‐minute walk test^47^), suggesting that their limitations were predominantly caused by AS rather than other factors not addressed by TAVR.

**Table 2:** Patient selection

	Key question	Red flags
Short‐term risks	Is the patient at risk for a major procedural complication?	High TAVR risk score Comorbidity‐complication dyads Technically complex procedure
Mid‐term recovery	Is the patient likely to return home and recover function following TAVR?	Physical frailty Poor social support Active depression
Long‐term benefits	Is the patient likely to gain meaningful longevity and quality of life from TAVR?	Advanced dementia Bedbound Cachexia or severe sarcopenia Disability for all or most ADLs End‐stage kidney, liver, lung disease
Patient preference	Does the patient understand the expected benefits/risks and want to proceed?	Limited comprehension Unrealistic expectations External pressure to proceed

## 5 POSTPROCEDURAL CARE OF NONAGENARIANS

Despite appropriate selection and technical execution, 30%‐40% of older patients report persistently poor functional status and quality of life following TAVR—especially frail older patients who were deconditioned before and increasingly‐so after the procedure. Therefore, frailty should not only be viewed as a prognostic marker, but also a therapeutic target than can be improved with exercise and nutrition, ideally in the context of a structured cardiac rehabilitation program.^48^ Nonagenarians have been shown to benefit from cardiac rehabilitation^49^ and should not be excluded but rather preferentially referred following TAVR. Cardiac rehabilitation programs can be adapted to their abilities and progress at a suitable pace; they can even be delivered in the home‐based setting as in the ongoing PERFORM‐TAVR Trial (Protein and Exercise for Reversal of Frailty in OldeR Men and women undergoing TAVR; NCT 03522454).

## 6 CONCLUSIONS

As the population ages, clinicians will encounter a growing number of nonagenarians suffering from severe AS. By virtue of a healthy survivor effect (or a referral bias), these patients can paradoxically have greater resilience and fewer comorbidities. They can, on average, tolerate the TAVR procedure quite well with low in‐hospital and 1‐year adverse event rates; acknowledging that this subgroup of patients is under‐represented or at the very least highly selected in randomized trials, and that the evidence to‐date stems mostly from observational studies. Appropriate patient selection should consider individualized estimates of procedural risk, potential for functional recovery and for improved quantity and quality of life. Age alone is inadequate to stratify patients. Frailty is much more revealing, and it can be measured by brief tools such as the EFT, and in selected cases by deeper evaluations such as that offered by a comprehensive geriatric assessment. Ultimately, the process of shared decision‐making is paramount to ensure that the course of action is patient‐centered and balances the procedure's expected risks and benefits with the nonagenarian's preferences and values.

## CONFLICT OF INTEREST

The authors declare no potential conflict of interests.
